# Online technical training course to train professionals who work with Chemical Dependency, promoted by a psychiatric clinic in Rio de Janeiro

**DOI:** 10.1192/j.eurpsy.2023.1824

**Published:** 2023-07-19

**Authors:** V. Soares De Jesus

**Affiliations:** Therapist Course, Clínica Jorge Jaber, Rio de Janeiro, Brazil

## Abstract

**Introduction:**

Description of a job of training technical professionals who have personal experience with Chemical Dependency to work in the area and resocialization of the mentally ill in order to combat social stigma.

**Objectives:**

Training technical professionals who, having gone through the experience of the disease, wish to help others and by participating in the training course for therapists in chemical dependence, they will seek to combat stigma.

**Methods:**

The Chemical Dependency Therapist Training course is a free course and takes place online, through a platform on a social network, which allows students to access it at any time of the day and can access it from outside Brazil.In the course students receive daily communication from the educational deparmtent. All patients during the hospitalization period are invited to attend the course, write down their doubts with the course professors.

**Results:**

The creation of a free online course, which has patient students and enrolled students from Brazil and the world, will help to train more professionals capable of working with chemical dependency.

**Conclusions:**

The online course has 1,800 students enrolled in 2022. These are students from Brazil and abroad, such as Portugal, France, Italy, Angola, United Kingdom, Finland, who are developing more knowledge and becoming professionals in the area of chemical dependence.

**Disclosure of Interest:**

None Declared

